# Determinants of Outcomes in Convulsive Status Epilepticus: An Observational Study at a Tertiary Care Center in North India

**DOI:** 10.7759/cureus.60017

**Published:** 2024-05-10

**Authors:** Ahmad G Ansari, Lubna Zafar, Ruhi Khan, Ariba Nasar

**Affiliations:** 1 Department of Medicine, Jawaharlal Nehru Medical College, Aligarh Muslim University, Aligarh, IND

**Keywords:** status epilepticus severity score, tertiary care centers, observational cross-sectional study, in-hospital outcome, north india, convulsive status epilepticus

## Abstract

Objective

Status epilepticus (SE) presents a critical neurological emergency associated with high morbidity and mortality rates worldwide. However, the determinants influencing outcomes in SE within specific regional contexts remain less explored, especially within North India. Understanding the factors influencing the prognosis of SE in this region is crucial for tailored therapeutic approaches and improved patient outcomes.

Materials and methods

This observational study was conducted at Jawaharlal Nehru Medical College, Aligarh, India, from December 1, 2020, to November 31, 2022. Patients who presented with convulsive SE lasting more than five minutes or repetitive and discrete seizures with impaired consciousness between the interictal period for at least 30 minutes were included in the study. Their clinical and biochemical variables at presentation were assessed and correlated with the outcome.

Results

Out of the 110 patients included in the study, males represented 59.1% (n=65), outnumbering females, who comprised 40.9% (n=45). Favourable outcome was observed in 66.36% (n=73) of patients, and unfavourable outcome was observed in 33.63% (n=37). The mean time interval between seizure onset to the patient’s arrival at the hospital was 5.30 ± 4.96 hours, and the mean time interval between seizure onset to the point of seizure control was 7.10 ± 6.38 hours. On analysing the factors associated with unfavourable outcome, the type of seizure at onset (p=0.021), Glasgow Coma Scale (GCS) of <=12 at presentation (p<0.001), presence of refractory seizure (p<0.001), presence of abnormal epileptiform discharges on electroencephalography (p=0.001), Status Epilepticus Severity Score (STESS) of >2 (p<0.001), serum lactate levels (p<0.001), duration of hospital stay (p=0.004), time interval between seizure onset to hospital arrival (p<0.001) and time interval between seizure onset to the point of seizure control (p<0.001) showed significant association. However, on analysing the independent risk factors of unfavourable outcome using multivariate logistic regression, only duration of hospital stay (p<0.001, odds ratio (OR): 1.205, 95% confidence interval (CI): 1.046-1.389), and GCS of less than or equal to 12 at presentation (p<0.001, OR: 12.354, 95% CI: 2.974-51.319) showed significant association.

Conclusions

Our study highlighted key clinical and time-related parameters influencing the outcome of convulsive SE. Understanding these factors is crucial for better treatment and improved patient outcomes. Further research is essential for refining interventions in this complex condition.

## Introduction

Status epilepticus (SE) is a neurological emergency necessitating intensive management. In SE, permanent brain damage is time-dependent, and the only prognostic indicator that can be influenced by treatment is the duration of seizure length [1]. While SE is a global concern, its impact can vary based on regional factors, such as demographics, healthcare infrastructure, and prevalent aetiologies. In the context of North India, where the burden of neurological disorders is substantial and diverse, SE poses a unique challenge. This region faces a combination of viral, metabolic, and structural elements that contribute to seizures, which are further impacted by socioeconomic circumstances, cultural norms, and healthcare accessibility [2]. However, studies specifically focusing on the determinants influencing SE outcomes within this geographical area are limited [3,4].

Understanding the determinants impacting SE outcomes in North India is crucial for several reasons. Firstly, the region's diverse demographic profile and varying healthcare accessibility can influence the timely administration of appropriate interventions, potentially affecting patient prognosis. Secondly, unique regional factors such as the prevalence of specific infectious diseases and genetic predispositions could play a role in the etiology and management of SE [5,6]. Exploring these factors within a tertiary care centre setting allows for a comprehensive analysis encompassing patient demographics, clinical presentations, treatment modalities, and associated complications. The aim of this study is to correlate clinical and biochemical parameters that determine the outcome in patients presenting with convulsive SE to provide a detailed insight into the management of SE tailored to the regional context, ultimately aiding in optimizing therapeutic strategies, improving healthcare delivery, and enhancing patient outcomes.

## Materials and methods

This observational study was conducted at the Department of Medicine, Jawaharlal Nehru Medical College A.M.U., a tertiary care centre in Aligarh, Uttar Pradesh, North India. This study was conducted between 1 December 2020 and 31 November 2022, over a period of two years. After taking informed consent, eligible patients were included in the study. Their clinical and biochemical parameters at presentation were recorded. The patients received the medical treatment as per the Department Convulsive Status Epilepticus treatment protocol adopted and modified from the International League against Epilepsy Guidelines, and their outcome was assessed [7]. The study protocol was approved by the Institutional Ethics Committee, Faculty of Medicine, Aligarh Muslim University, Aligarh, India (IEC NO: IECJNMC/503), and the study was conducted as per the standards of Good Clinical Practice and the Helsinki Declaration.

Study procedure

The study included patients who were above the age of 14 years and presented at the Emergency Trauma Centre or patients who were already admitted to the Medicine ward, Critical Care Ward, or Intensive Care Unit (ICU) with convulsive seizures lasting more than five minutes or repetitive and discrete seizures with impaired consciousness between the interictal period for at least 30 minutes. Patients with acute traumatic head injury, myoclonic epilepsies, psychogenic seizures, brain tumours/post-neurosurgery seizures, and eclampsia and patients or patient's attendants who denied consent were excluded from the study. After written informed consent was obtained, the following data were collected and recorded in an Excel sheet.

Patient parameters

The demographic parameters that were included were age, gender, and past history of epilepsy, which were recorded by a questionnaire. The types of seizure at presentation, as defined by Fisher et al. [8], that is, generalised tonic-clonic seizures (GTCS), focal seizures with secondary generalization (FBTCS), or uncertain to tonic clinic (UTCS), were recorded. The Glasgow Coma Scale (GCS) at presentation and the presence of refractory seizure, that is, SE that does not cease with the administration of two antiseizure medications, were noted [7]. The mean arterial pressure at presentation, Status Epilepticus Severity Score (STESS) at presentation, and duration of hospital stay were also noted. Biochemical parameters at presentation that were included in the study were random blood glucose, blood urea nitrogen, serum creatinine, total leucocyte count (TLC), serum lactate, pH, serum electrolytes (sodium, potassium, calcium, magnesium), and cerebrospinal fluid (CSF) analysis. Each patient underwent an electroencephalogram (EEG) examination, during which the presence or absence of epileptiform abnormalities on the EEG was recorded. Etiology was determined based on history, clinical examination, relevant investigations, and neuroimaging.

Time-Related Parameters Recorded at Presentation: T1A and T2C

In our study, T1A represents the time interval, measured in hours, between the start of a new seizure episode of convulsive SE and the patient's arrival at the medical facility. T2C, on the other hand, represents the time interval, also measured in hours, between the onset of a seizure and the cessation of tonic-clonic activity and improvement in consciousness.

Outcomes

The outcome of patients under study was categorized into patients with 1) favourable outcome, that is, patients who were discharged after cessation of seizures and recovered fully without any neurological deficit or impaired consciousness; and 2) unfavourable outcome, that is, patients who were discharged but still had residual neurological deficit or impaired consciousness due to complications of convulsive SE or the primary disease itself, as well as those who expired.

Statistical analysis

The presentation of the categorical variables was done in the form of numbers and percentages (%). On the other hand, the quantitative data with normal distribution were presented as the means ± standard deviation (SD). In the cases in which the data were not normal, we used nonparametric tests. The following statistical tests were applied for the results: 1) the association of the variables that were quantitative and not normally distributed in nature was analysed using the Mann-Whitney test, and the variables that were quantitative and normally distributed in nature were analysed using the independent t-test. 2) The association of the variables that were qualitative in nature was analysed using the chi-square test. If any cell had an expected value of less than 5 then Fisher’s exact test was used. 3) Univariate and multivariate logistic regression was used to find out significant risk factors of unfavourable outcomes. 4) Receiver operating characteristic (ROC) curve was used to find the cutoff point, sensitivity, specificity, positive predictive value, and negative predictive value of age (T1A (hours) and T2C (hours)) and STESS at presentation for predicting unfavourable outcomes. Data entry was done in the Microsoft Excel spreadsheet, and the final analysis was done with the use of Statistical Product and Service Solutions (SPSS, version 25.0; IBM SPSS Statistics, Armonk, NY) software. For statistical significance, a p value of less than 0.05 was considered statistically significant.

## Results

**Figure 1:**
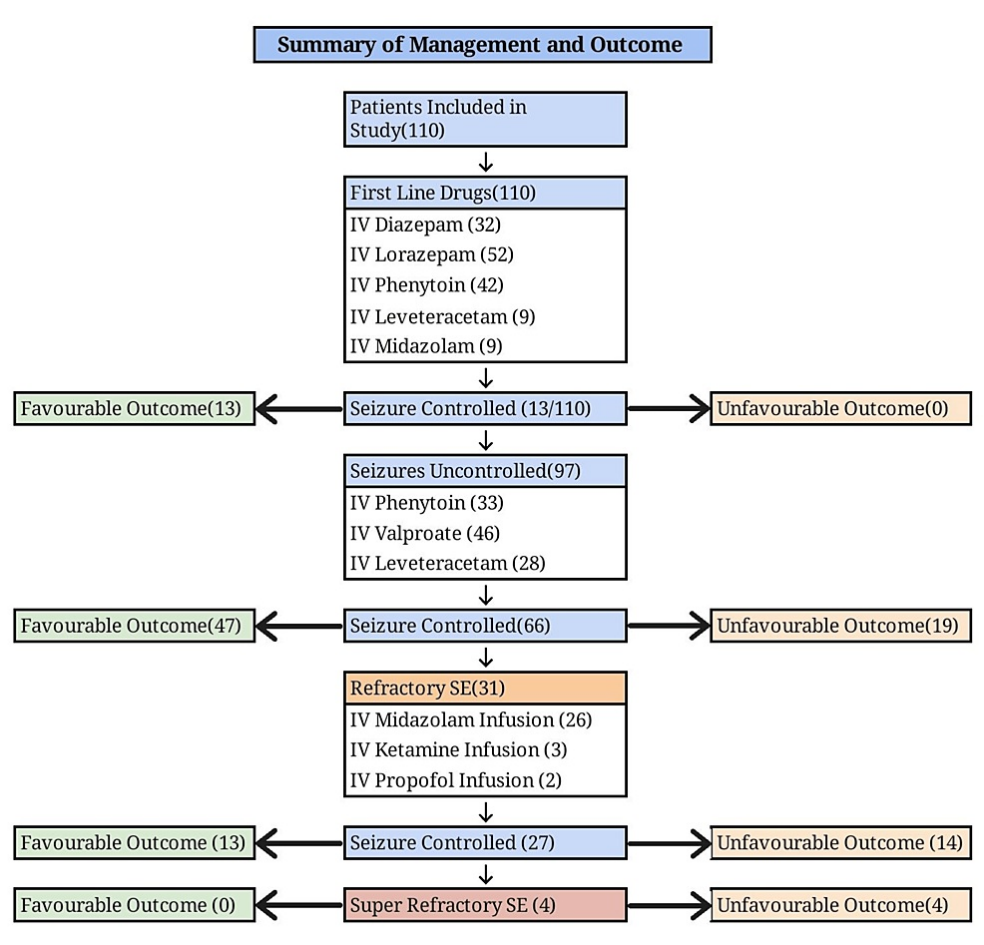
Summary of treatment and outcome among 110 patients included in the study IV: Intravenous, SE: Status Epilepticus

The study was conducted from December 1, 2020, to November 31, 2022. We included 110 patients who presented with convulsive SE and met our inclusion criteria. The outcome of the study is summarized in Figure 1.

Out of the 110 patients included in the study, 66.4% (n=73) of the patients had favourable outcomes, and 33.6% (n=37) had unfavourable outcomes. Out of them, 23.6% (n=26) were discharged but still had residual neurological deficits or impaired consciousness, and 10% (n=11) of patients expired.

Demographic variables versus outcomes

**Figure 2:**
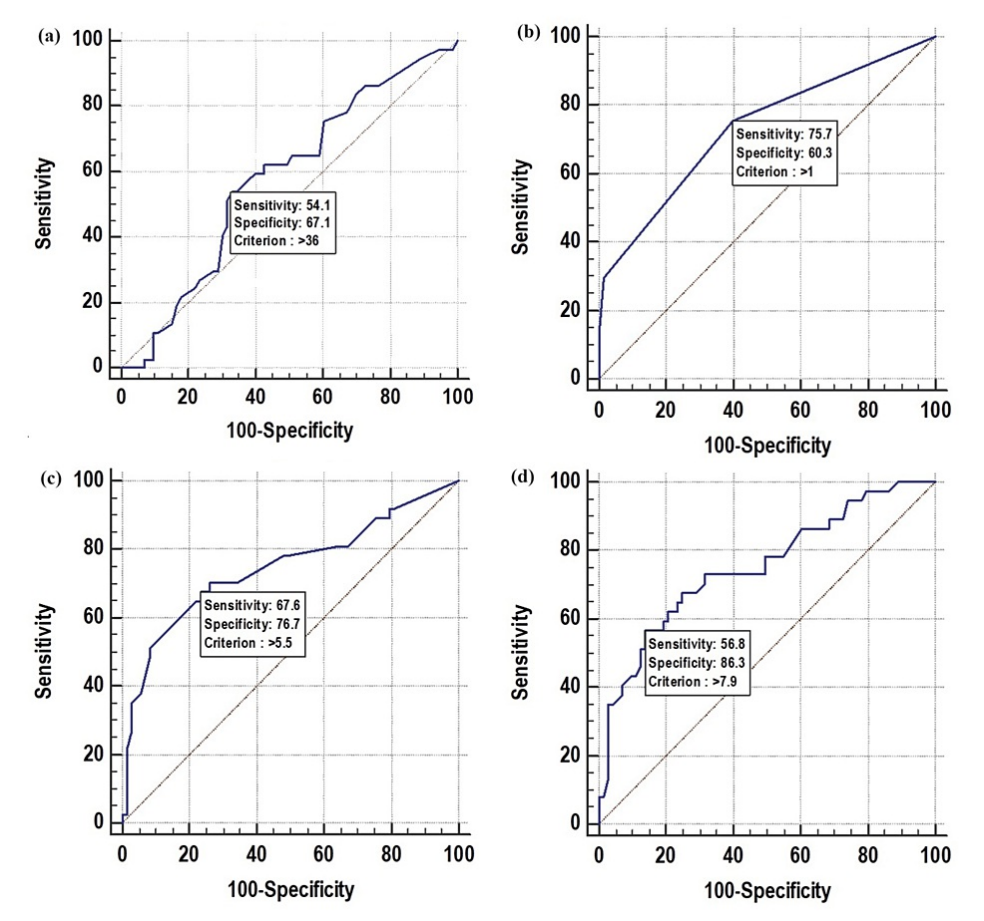
Receiver operating characteristic curve of age (a), STESS at presentation (b), T1A (hours) (c), and T2C (hours) (d) for predicting unfavourable outcomes

The mean age of the patients included in our study was 35.7 ± 17.9 years. Upon further analysis, age was examined as a predictor of unfavourable outcomes. The ROC curve for age revealed an area under curve (AUC) value of 0.579. The cutoff criterion for age was determined to be greater than 36 years (p=0.162, sensitivity: 54.05%, specificity: 67.12, positive predictive value (PPV): 45%, and negative predictive value (NPV): 74.2%) (Table 1, Figure 2). Using a 36-year age threshold, we investigated the relationship between age and unfavourable outcomes. Among patients aged over 36 years (40%, n=44), 45.45% (n=20) experienced unfavourable outcomes, and a significant association between age and unfavourable outcomes was observed (p=0.032; Table 2). However, when evaluating age as an independent factor of unfavourable outcomes using multivariate logistic regression, no significant association was observed (p=0.844, odds ratio (OR): 1.149, 95% confidence interval (CI): 0.286-4.68) (Table 3). The association of gender with unfavourable outcomes was also not statistically significant (p=0.444; Table 2).

**Table 1:** Receiver operating characteristic curve of age, T1A (hours), T2C (hours), and STESS at presentation for predicting unfavourable outcomes NPV: Negative predictive value; PPV: Positive predictive value; ROC: Receiver operating characteristic; STESS: Status epilepticus severity score; T1A: Time interval (in hours) between the start of a new seizure episode of convulsive status epilepticus and the patient's arrival at the medical facility; T2C: Time interval (in hours) between the onset of a seizure and the cessation of tonic-clonic activity and improvement in consciousness

Variables	Area under the ROC curve (AUC)	P value	Cutoff	Sensitivity	Specificity	PPV	NPV
Age (years)	0.579	0.162	>36	54.05%	67.12%	45.50%	74.20%
T1A (hours)	0.748	<0.001	>5.5	67.57%	76.71%	59.50%	82.40%
T2C (hours)	0.753	<0.001	>7.9	56.76%	86.30%	67.70%	79.70%
STESS at presentation	0.735	<0.001	>1	75.68%	60.27%	49.10%	83%

**Table 2:** Demographic variables and their association with the outcome Data presented as frequency (n) and percentage (%) † Chi-square test

Parameters	-	Total (n=110)	Favourable Outcome (n=73)	Unfavourable Outcome (n=37)	P
Age (Years)	<=36	66 (60%)	49 (74.24%)	17 (25.76%)	0.032†
-	>36	44 (40%)	24 (54.54%)	20 (45.45%)	-
Gender	Male	65 (59.1%)	45 (69.23%)	20 (30.77%)	0.444†
-	Female	45 (40.9%)	28 (62.22%)	17 (37.78%)	-

**Table 3:** Multivariate logistic regression to find out independent significant risk factors of an unfavourable outcome Variables presented as odds ratio (95% confidence interval). OR: Odds ratio, CI: Confidence interval, GCS: Glasgow Coma Scale, GTCS: Generalised tonic-clonic seizure, FBTCS: Focal to bilateral tonic-clonic, UTCS: Uncertain to tonic-clonic, EEG: Electroencephalography, STESS: Status epilepticus severity score

Variables	OR (95% CI)	P value
Lactate (mmol/L)	1.3148 (0.941-1.863)	0.108
Duration of hospital stay (days)	1.205 (1.046-1.389)	<0.001
Age (Years)	-	-
>36	1.149 (0.286-4.68)	0.844
<=36	1 [Reference]	
Type of seizure at onset	-	-
GTCS	0.378 (0.075-1.887)	0.235
FBTCS	1.242 (0.126-12.196)	0.852
UTCS	1 [Reference]	-
GCS at presentation	-	-
<=12	12.354 (2.974-51.319)	<0.001
>12	1 [Reference]	-
Refractory seizure	-	-
Absent	0.265 (0.064-1.098)	0.067
Present	1 [Reference]	-
Epileptiform abnormalities on EEG	-	-
Absent	2.66 (0.649-10.971)	0.174
Present	1 [Reference]	0.226
STESS at presentation	-	-
>2	13.625 (0.759-244.381)	0.076
<=2	1 [Reference]	-
T1A(hours)	-	-
>5 hours	7.829 (0.558-109.765)	0.126
<=5 hours	1 [Reference]	0.182
T2C(hours)	-	-
>5 hours	1.14 (0.286-4.618)	0.844
<=5 hours	1 [Reference]	-

Clinical and biochemical variables versus outcomes

The mean arterial blood pressure (MAP) at presentation observed in our study was 88.21 ± 21.7 mmHg, and 38.46% of patients with MAP of less than 80 mmHg at presentation had unfavourable outcomes as compared to 30.99% with MAP of more than 80 mmHg at presentation. The most common type of seizure at presentation observed in our study was GTCS (65.45%, n=72). UTCS was observed in only 25.45% (n=28) of patients, with 53.57% (n=15) of patients with UTCS having unfavourable outcomes (Table 4). The association of the type of seizure at presentation with unfavourable outcomes was analysed and showed a statistically significant result (p=0.021). In our study, we observed refractory seizures in 28.18% of patients, and the presence of refractory seizures was significantly associated with poor outcomes (p<0.001) (Table 4). However, upon further analysis, the presence of refractory seizures did not show a significant association as an independent risk factor of unfavourable outcomes (p=0.067, OR: 0.265, 95% CI: 0.064-1.098) (Table 3). A significant association was also observed between a GCS score of less than or equal to 12 at presentation and unfavourable outcomes (p<0.001). Among patients without refractory seizures, only 24.05% experienced an unfavourable outcome, while 58.06% of those with refractory seizures had a unfavourable outcome. Similarly, a significant association was observed between the presence of EEG findings of epileptiform abnormalities and unfavourable outcomes, with 43.4% of patients with epileptiform abnormalities having unfavourable outcomes, as compared to only 13.51% of patients without epileptiform abnormalities on EEG (p=0.001) (Table 4). In our study, the average length of hospitalisation was 9.42 ± 6.22 days. Furthermore, a statistically significant association was observed between the duration of hospital stay and unfavourable outcome (p=0.004) (Table 5).

**Table 4:** Clinical characteristics and their association with the outcome Data presented as frequency (n) and percentage (%); CSF: Cerebrospinal fluid, FBTCS: Focal to bilateral tonic-clonic seizures, GCS: Glasgow coma scale, GTCS: Generalised tonic-clonic seizures, EEG: Electroencephalogram, MAP: Mean arterial pressure, RBS: Random blood sugar, STESS: Status epilepticus severity scale, SD: Standard deviation, UTCS: Uncertain to tonic-clonic seizures ‡ Independent t-test, *Fisher's exact test, † Chi-square test

Parameters	-	Total (n=110)	Favourable Outcome (n=73)	Unfavourable Outcome (n=37)	P
Known case of epilepsy	Present	21 (19.09%)	15 (71.43%)	6 (28.57%)	0.585†
-	Absent	89 (80.09%)	58 (65.17%)	31 (34.83%)	-
Etiology	Acute Symptomatic Etiology	68 (61.82%)	41 (60.29%)	27 (39.71%)	0.086†
-	Others	42 (38.18%)	32 (76.19%)	10 (23.81%)	-
Type of seizure as per onset	GTCS	72 (65.45%)	54 (75%)	18 (25%)	0.021*
-	FBTCS	10 (9.09%)	6 (60%)	4 (40%)	-
-	UTCS	28 (25.45%)	13 (46.43%)	15 (53.57%)	-
GCS at presentation	>12	66 (60%)	58 (87.88%)	8 (12.12%)	<0.001†
-	<=12	44 (40%)	15 (34.09%)	29 (65.91%)	-
Refractory seizure	Present	31 (28.18%)	13 (41.94%)	18 (58.06%)	<0.001†
-	Absent	79 (71.82%)	60 (75.95%)	19 (24.05%)	-
MAP at presentation (mmHg)	>=80	71 (64.54%)	49 (69.01%)	22 (30.99%)	0.427†
-	<80	39 (35.45%)	24 (61.54%)	15 (38.46%)	-
RBS at presentation (mg/dL)	>=140	23 (20.91%)	16 (69.57%)	7 (30.43%)	0.066†
-	70 to 140	60 (54.54%)	44 (73.33%)	16 (26.67%)	-
-	<70	27 (24.54%)	13 (48.15%)	14 (51.85%)	-
Epileptiform abnormalities on EEG	Absent	37 (33.64%)	32 (86.49%)	5 (13.51%)	0.001†
-	Present	73 (66.36%)	41 (56.16%)	32 (43.84%)	-
STESS at presentation	>2	12 (10.91%)	1 (8.33%)	11 (91.67%)	<0.001*
-	<=2	98 (89.09%)	72 (73.47%)	26 (26.53%)	-
CSF analysis	Normal	69 (62.73%)	49 (71.01%)	20 (28.99%)	0.18†
-	Abnormal	41 (37.27%)	24 (58.54%)	17 (41.46%)	-

**Table 5:** Analysis of quantitative variables and their association with the outcome Data presented as mean ± SD (standard deviation) ‡ Independent t-test

Parameters	Mean ± SD	Favourable Outcome (n=73)	Unfavourable Outcome (n=37)	P
Lactate (mmol/L)	5.8 ± 2.42	5.08 ± 1.84	7.22 ± 2.79	<0.001 ‡
S. creatinine (mg/dL)	1.93 ± 3.76	1.87 ± 3.58	2.07 ± 4.16	0.797 ‡
Duration of hospital stay (days)	2 ± 6.22	8.20 ± 4.43	11.81 ± 8.30	0.004 ‡

The STESS with an unfavourable outcome was also significant, with 26.53% of patients with a STESS of 2 or lower experiencing an unfavourable outcome, while 91.67% of patients with a STESS greater than 2 had an unfavourable outcome (p<0.001). The ROC analysis of the STESS at presentation with an unfavourable outcome revealed an AUC of 0.735, with a cutoff score of more than 1 (p<0.001, sensitivity: 75.6%, specificity: 60.2%, PPV: 49.1%, and NPV: 83%) (Table 1, Figure 2). Mean values of biochemical variables included in our study are shown in Table 6. The mean serum lactate levels of patients upon initial presentation were 5.8 ± 2.4 mmol/L. There was a notable correlation between the mean serum lactate levels and unfavourable outcomes, which was statistically significant (p<0.001) (Table 5).

**Table 6:** Biochemical investigations performed Data presented as mean ± SD (standard deviation)

Investigations	Mean ± SD
Random blood glucose at presentation (mg/dL)	106.55 ± 66.77
Serum sodium (mEq/dL)	137.71 ± 8.84
Total leucocyte count (x10³/mm³)	14.38 ± 8.87
pH	7.23 ± 0.15
Blood urea nitrogen (mg/dL)	27.54 ± 31
Lactate (mmol/L)	5.8 ± 2.42
Serum potassium (mEq/dL)	4.04 ± 1.03
Serum calcium (mg/dL)	8.13 ± 1.28
Serum magnesium (mg/dL)	1.84 ± 0.41
Serum creatinine (mg/dL)	1.94 ± 3.77

Timed variables versus outcomes

The mean duration of timed variables with clinical parameters is shown in Table 7. The mean duration of the time interval between the onset of seizures and the arrival of the patient at the hospital (T1A) observed in our study was 5.3 ± 4.96 hours, while the mean duration of the time interval between seizure onset and cessation of clinical seizures (T2C) was 7.1 ± 6.38 hours. A statistically significant association was observed between the mean duration of T1A and an unfavourable outcome (Table 8), with the mean duration of T1A in patients with an unfavourable outcome being 8.26 ± 5.94 hours, as compared to 3.8 ± 3.5 hours in patients with a favourable outcome (p<0.001). A similar association was observed in patients who presented within five hours of the onset of convulsive SE, with only 18.18% having an unfavourable outcome as compared to 56.82% in patients who presented after five hours of the onset of convulsive SE (p<0.001). Similarly, a significant statistical association between T2C and an unfavourable outcome was observed, with a duration of more than five hours for cessation of seizures associated with an unfavourable outcome (p<0.001) (Table 8). The ROC analysis of the T1A and T2C with an unfavourable outcome revealed an AUC of 0.748 (p<0.001) and 0.753 (p<0.001), respectively. The cut-off for T1A was observed to be more than 5.5 hours and for T2C more than 7.9 hours (Table 1, Figure 2).

**Table 7:** Mean duration of timed variables with clinical parameters Data presented as mean ± SD (standard deviation) in hours. AED: Antiepileptic drug, CNS: Central nervous system, GCS: Glasgow coma scale, MAP: Mean arterial pressure

Parameters		T1A (Hours)		T2C (Hours)	
-	-	Favourable Outcome	Unfavourable Outcome	Favourable Outcome	Unfavourable Outcome
Age	>36 years	3.86 ± 3.39	8.03 ± 5.57	4.73 ± 3.47	12.2 ± 9
-	<=36 years	3.78 ± 3.7	8.47 ± 6.38	5.29 ± 4.76	10.05 ± 6.74
Gender	Male	3.98 ± 3.77	8.65 ± 6.15	5.47 ± 4.71	11.41 ± 8.51
-	Female	3.52 ± 3.31	7.82 ± 5.85	4.53 ± 3.81	10.60 ± 7.16
Known case of epilepsy	Present	4.26 ± 2.18	12.33 ± 7.87	4.84 ± 2.27	16.88 ± 10.30
-	Absent	3.69 ± 3.87	7.48 ± 5.31	5.18 ± 4.79	9.91 ± 6.90
MAP	>=80 mmHg	3.63 ± 3.9	7.14 ± 4.65	4.81 ± 4	9.43 ± 7.16
-	<80 mmHg	4.17 ± 2.87	9.93 ± 7.31	5.73 ± 5.1	13.4 ± 8.39
Etiology	AED noncompliance	4.6 ± 2.12	15 ± 8.66	5.29 ± 2.18	21.37 ± 9.57
-	CNS infections	3.95 ± 4.63	9.88 ± 4.86	5.67 ± 6	11.81 ± 6.19
-	Vascular	2 ± 2.31	6.3 ± 5.56	3.03 ± 2.01	9.01 ± 8.06
-	Metabolic Abnormality	1.62 ± 2.22	6.75 ± 6.99	3.01 ± 3.01	12 ± 9.8
-	Demyelinating	-	6 ± 0	-	6.75 ± 0
-	Autoimmune	3 ± 0	0.9 ± 0	5 ± 0	2 ± 0
-	Unknown	5.52 ± 2.79	3 ± 1.41	6.58 ± 3.18	4.25 ± 2.47
GCS at presentation	>12	3.97 ± 3.73	6.25 ± 2.96	5.39 ± 4.67	8.86 ± 8.11
-	<=12	3.17 ± 2.99	8.83 ± 6.46	4.04 ± 2.87	11.64 ± 7.78

**Table 8:** Association between timed variables and the outcome Data presented as frequency (n) and percentage (%). ‡ Independent t-test, † Chi-square test

Parameters	-	Total (n=110)	Favourable Outcome (n=73)	Unfavourable Outcome (n=37)	P
T1A	Mean ± SD (hours)	5.30 ± 4.96	3.80 ± 3.58	8.26 ± 5.94	<0.001‡
-	<=5 hours	66	54 (81.82%)	12 (18.18%)	<0.001†
-	>5 hours	44	19 (43.18%)	25 (56.82%)	-
T2C	Mean ± SD (hours)	7.10 ± 6.38	5.11 ± 4.38	11.03 ± 7.82	<0.001‡
-	<=5 hours	60	50 (83.33%)	10 (16.67%)	<0.001†
-	>5 hours	50	23 (46%)	27 (54%)	-

Of the variables with a significant association with an unfavourable outcome, independent risk factors of an unfavourable outcome were analysed with multivariate logistic regression, and it was observed that only the duration of hospital stay (p<0.001, OR: 1.205, 95% CI: 1.046-1.389) and a GCS score of less than or equal to 12 (p<0.001, OR: 12.345, 95% CI: 2.974-51.319) were significant independent risk factors for an unfavourable outcome (Table 3).

## Discussion

Our study observed that 33.6% of the patients had an unfavourable result, while only 10% of the patients in the study expired. A wide range of mortality in convulsive SE has been reported from 10% to 39%, which may be attributed to variations in the characteristics of the study population [6,9,10]. In our study, a significant association with an unfavourable outcome was observed with age more than 36 years, a GCS of less than or equal to 12 at presentation, the presence of refractory seizures, the type of seizures at presentation, the presence of epileptiform abnormalities on EEG, the STESS of more than 2 at presentation, serum lactate levels, duration of hospital stay, and time interval between seizure onset to hospital arrival and time interval between seizure onset to the point of seizure control.

Demographic variables versus outcomes

The average age of presentation in the study population was 35.7 ± 17.9 years, ranging from 14 to 75 years. Older age at presentation was associated with poor outcomes, and a similar association has been shown in past studies. The cutoff of age with poor outcomes in our study was 36 years; however, the association was not significant [2,3,11-14]. While some studies have shown female gender being associated with unfavourable outcomes, the gender of our study patients did not show any significant correlation, although male patients were more in number than female patients [15,16].

Clinical variables versus outcomes

The most common etiology for SE observed in our study was due to CNS infections (40%, n=44), followed by antiepileptic drug noncompliance (16.4%, n=18), metabolic abnormalities (15.5%, n=17), vascular causes (ischemic and haemorrhagic stroke and cavernous sinus thrombosis) (12.7%), and unknown etiology (12.7%). An intricate interplay between genetic factors and the environment has been shown to have a role in the early development of epilepsy. Furthermore, the causes of SE vary significantly depending on the geographical location [17]. In their retrospective study, Li et al. reported that CNS infections were the most prevalent aetiology in the Chinese population, whereas Leitinger et al. reported that cerebrovascular causes were the most common form of aetiology in their own retrospective study [18,19]. The most common type of seizure at presentation in our study was GTCS, while patients with UTCS had the most unfavourable outcome [6]. This is in contrast to the ambispective study conducted by Dani et al., who reported GTCS as a determinant for unfavourable outcomes [3]. A low GCS score at the time of the first assessment (GCS of <=12 in our study) was identified as a significant independent risk factor for an unfavourable outcome. Numerous previous research has shown similar findings [1,11,20-22]. A higher incidence of unfavourable outcomes was seen in individuals with a MAP below 80 mmHg, accounting for 38.46% of cases. Dani et al. conducted a prior study that related MAP to presentation and outcomes in patients with convulsive SE [3]. Previous studies have shown a significant association between STESS and unfavourable outcomes [10,21,23,24]. This finding aligns with the results of our study, showing a significant association between a STESS of more than 2 and an unfavourable outcome in study patients. However, our study did not observe an independent association between STESS and poor outcome (p=0.127). The results of our investigation revealed a significant association between the levels of serum lactate at initial presentation and a dismal outcome, which is consistent with previous studies [25,26]. The presence of refractory seizures demonstrated a significant correlation with unfavourable outcomes (p<0.001). The research on refractory SE and unfavourable outcomes has been the focus of extensive research. Several studies have investigated the epidemiology, risk factors, and outcomes of refractory seizures [12,27,28]. The average duration of hospitalisation for the patients in our study was 9.42 ± 6.22 days, and a statistically significant correlation was observed with unfavourable outcomes (p=0.004). In our study, we observed that the duration of hospitalisation was an independent significant risk factor associated with an unfavourable outcome (p<0.001, OR: 1.205, 95% CI: 1.046-1.389). The results were consistent with the findings of Kenichiro Sato et al., who observed a median hospital stay of 10 days (with an interquartile range of 4-19 days) in their retrospective analysis. Furthermore, a longer duration of hospital stay was observed to be associated with a negative outcome [29].

Timed variables versus outcomes

A significant correlation was observed between the time to arrive at the hospital (T1A) and the time it took to control the seizure (T2C), resulting in an unfavourable outcome. A systemic analysis conducted by Neligan et al. reported that a longer duration of SE was correlated with a negative outcome [30]. In our study, we determined that the cutoff for T1A in ROC analysis was 5.5 hours, while for T2C, it was 7.9 hours. The corresponding AUC values were 0.748 and 0.753, respectively. However, neither of these variables was found to be independent risk factor for an unfavourable outcome. In their prospective study, Murthy et al. reported that the average duration between the onset of SE and hospital treatment was 18.02 hours, with a range of 1-72 hours. They also reported that a longer duration was associated with a poor outcome (p=0.001) [4]. In a recent ambispective observational study, Dani et al. reported that the cutoff for the time to seek medical attention was 3.5 hours (AUC: 0.79), while the time for seizure control from the onset of seizure was also 3.5 hours (AUC: 0.69). However, only the time to seek medical attention was identified as an independent risk factor for an unfavourable outcome [3].

Limitations of the study

It is important to acknowledge the limitations of the present study design in order to properly understand the findings of this observational study. Firstly, the study is constrained by a relatively small sample size, which may limit the generalizability of the results to broader populations. Additionally, the lack of longitudinal follow-up restricts the comprehensive understanding of the long-term outcomes and potential prognostic factors associated with convulsive SE. Furthermore, the complexity of SE warrants multifaceted investigations, suggesting a need for further research endeavors encompassing larger cohorts and extended follow-up periods to elucidate the intricate interplay of variables influencing the outcomes of convulsive SE more comprehensively. Thus, while the present study contributes valuable insights, these limitations underscore the necessity for future research initiatives to refine our understanding of convulsive SE management and prognostication effectively.

## Conclusions

In conclusion, our observational study within a North Indian tertiary care centre reveals a significant correlation between a GCS score of less than or equal to 12 and a longer duration of hospital stay with poor outcomes in patients with SE. The prognosis becomes increasingly unfavourable as the time taken to arrive at the hospital and the duration of seizures increases. These findings underscore the critical role of early neurological assessment and prompt intervention in improving prognosis. Recognizing the impact of GCS and hospital stay duration on outcomes provides actionable insights for healthcare practitioners, emphasizing the importance of tailored care strategies to mitigate the associated risks and enhance the overall management of SE in this regional setting.
